# An automated framework for 3D serous pigment epithelium detachment segmentation in SD-OCT images

**DOI:** 10.1038/srep21739

**Published:** 2016-02-22

**Authors:** Zhuli Sun, Haoyu Chen, Fei Shi, Lirong Wang, Weifang Zhu, Dehui Xiang, Chenglin Yan, Liang Li, Xinjian Chen

**Affiliations:** 1 School of Electronic and Information Engineering, Soochow University, Suzhou, Jiangsu, 215006, China; 2 Joint Shantou International Eye Center, Shantou University and the Chinese University of Hong Kong, Shantou, Guangdong, 515041, China; 3 College of Physics, Optoelectronics and Energy, Soochow University, Suzhou, Jiangsu, 215006, China

## Abstract

Pigment epithelium detachment (PED) is an important clinical manifestation of multiple chorioretinal diseases, which can cause loss of central vision. In this paper, an automated framework is proposed to segment serous PED in SD-OCT images. The proposed framework consists of four main steps: first, a multi-scale graph search method is applied to segment abnormal retinal layers; second, an effective AdaBoost method is applied to refine the initial segmented regions based on 62 extracted features; third, a shape-constrained graph cut method is applied to segment serous PED, in which the foreground and background seeds are obtained automatically; finally, an adaptive structure elements based morphology method is applied to remove false positive segmented regions. The proposed framework was tested on 25 SD-OCT volumes from 25 patients diagnosed with serous PED. The average true positive volume fraction (TPVF), false positive volume fraction (FPVF), dice similarity coefficient (DSC) and positive predictive value (PPV) are 90.08%, 0.22%, 91.20% and 92.62%, respectively. The proposed framework can provide clinicians with accurate quantitative information, including shape, size and position of the PED region, which can assist clinical diagnosis and treatment.

Optical coherence tomography (OCT) was first introduced in 1991 by Huang *et al.*^1^. Recently, spectral domain (SD) OCT has been used in diagnosis of many ocular diseases, including age-related macular degeneration (AMD), glaucoma and diabetic macular edema^2^. SD-OCT has many advantages comparing to traditional OCT, such as high resolution, real 3D volumetric image of retina and manifestation of more comprehensive anatomical structures. PED is an important feature of several chorioretinal diseases, such as, AMD, central serous chorioretinopathy and polypoidal choroidal vasculopathy^3^,^4^. PED can cause damage to central vision finally^5^,^6^. Generally, PED can be classified into three types: serous, drusenoid and fibrovascular. Though the three types of PED share several basic similarities, there are many distinct differences in clinical and prognostic aspects. For example, the serous PED tends to be a smooth, arch-like shape region with retinal pigment epithelium (RPE) deformation^3^. As shown in Fig. 1, the serous PED region is located between RPE floor and Bruch’s membrane (BM). Quantitative information of serous PED, including accurate boundary, size, position and total number, is important for diagnosis and treatment of the relevant retinal diseases. Therefore, automatic segmentation for serous PED objects in SD-OCT is of great clinical significance.

However, automatic segmentation for abnormal retinal structures still remains a challenging task. There are two critical problems for this task. First, the retinal morphology and intensity may have changed severely resulting from the abnormal structures. Therefore, the prior knowledge about morphological and optical features used for normal retinal image segmentation may not be valid. Second, the segmentation performance is affected by the blurred boundary, various shapes and random position of the abnormal structures. For retinal images analysis, an important pre-processing step for region segmentation is retinal layers segmentation. The retinal layers segmentation result can serve as constraints for automatic detection or segmentation of the abnormalities. Previously, many effective methods for automatic retinal layers segmentation on normal retinal images have been reported^7^,^8^,^9^,^10^,^11^. However, those segmentation methods tend to fail when the retina has serious deformation. Then some works aimed to segment abnormal retinal layers with relatively serious deformation of retinal structures^12^,^13^,^14^,^15^,^16^. The method in ref. 15 reported good performance of segmenting abnormal retinal layers, however, the accuracy of segmenting abnormal regions still can be improved.

Previously, some related works have been presented for detecting or segmenting retinal abnormalities. Fernández used a deformable model to roughly outline the fluid-filled regions from AMD patients, but its initialization result was obtained manually in 2D OCT images^17^. Ahlers *et al.* detected the location and measured the volume of the fibrovascular PED by using the high-definition OCT tool^18^. Quellec *et al.* detected automatically the 2D footprints of the symptomatic exudate-associated derangements (SEAD) in SD-OCT images by using a classification method, and used an interactive computer-aided method to define the 3D SEAD^19^. Dolejši *et al.* segmented semi-automatically the 3D SEADs associated with wet AMD by using a two-step segmentation method, and its initialization result was obtained manually^20^. Gregori *et al.* measured the drusen area and volume in eyes with non-exudative AMD from 74 patients by using the SD-OCT imaging^21^. Penha *et al.* measured the PED area and volume by using the Cirrus SD-OCT imaging system and its automatic measurement algorithm^22^. Wilkins *et al.* used a retinal cyst segmentation technique to segment cystoids fluid regions automatically in OCT images^23^. Chen *et al.* used an automated framework to segment 3D SEADs in macula-centered 3D OCT images^13^. Zheng *et al.* used an interactive segmentation method to quantify the intraretinal and subretinal fluids in retinal SD-OCT volume scans from 37 patients with exudative AMD^24^. Ding *et al.* detected the sub-retinal fluid and sub-retinal pigment epithelium fluid automatically by using a segmentation learning pipeline^25^.

However, although the above related works have been reported in recent years, the automatic segmentation for PED is still a challenging problem. The severe deformation, various shapes and random position of serous PED make automatic segmentation even more difficult. In this paper, we propose an automated framework to segment serous PED by effectively combining the multi-scale graph search, shape-constrained graph cut and mathematical morphology algorithm. The proposed framework can provide clinicians with accurate quantitative information, including shape, size and position of the PED region, which can assist diagnosis and treatment.

The contributions of this work are summarized as follows. (1) A novel automated framework is proposed for 3D serous PED segmentation in SD-OCT images, and the segmentation result demonstrates the efficiency and feasibility of the proposed framework. (2) The foreground and background seeds used in the shape-constrained graph cut algorithm are obtained automatically, which makes the proposed framework automatic. (3) An effective AdaBoost method is applied to remove false positive segmented regions in the initial segmentation results. (4) The mathematical morphology method is applied to refine the automatic segmentation result obtained from the shape-constrained graph cut method, in which the structure element is chosen adaptively.

## Results

### Dataset

The proposed framework was tested on a dataset of 25 SD-OCT images from 25 patients diagnosed with serous PED. Those SD-OCT images were obtained using the Topcon 3D-OCT 1000 (Topcon corporation, Tokyo, Japan). Each SD-OCT volume contains 512 × 64 × 480 voxels with a corresponding voxel size of 11.72 μm × 93.75μm × 3.50 μm.

### Automatic segmentation result of the proposed framework

**Figure 2:**
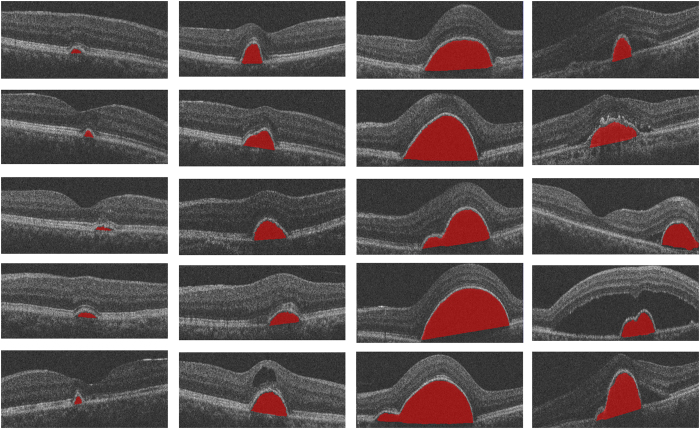
The final automatic segmentation results of serous pigment epithelium detachment in different subjects.

**Figure 3:**
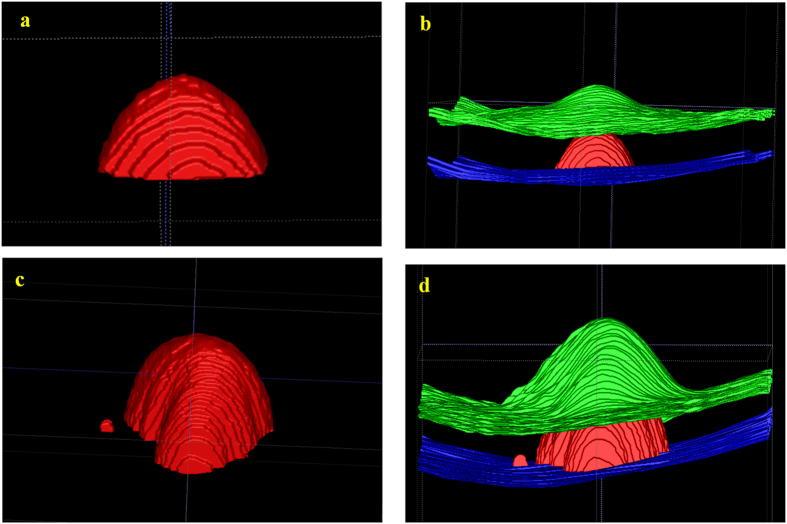
3D visualization of the serous pigment epithelium detachment segmentation results. The red regions represent the segmented serous pigment epithelium detachment, green surfaces represent the internal limiting membrane surface and blue surfaces represent the Bruch’s membrane surface.

Figure 2 shows the final automatic segmentation results on B-scans of different SD-OCT images. The first column shows small serous PED regions; the second column shows medium serous PED regions; the third column shows big serous PED regions; and the last column shows not only serous PED but also other co-existed fluid-associated abnormalities. The serous PEDs in the last column with other complications are more challenging to get accurate segmented regions, because those fluid-filled regions with similar intensity need to be distinguished from serous PED regions. Figure 3 shows two examples of the 3D visualization of the serous PED segmentation results, which provide stereoscopic information of serous PEDs for doctors.

### Comparison of segmentation performance

Table 1 shows the segmentation performance. The mean and standard deviation of TPVF, FPVF, DSC and PPV, defined by Equation (1)–(4), 2, 3, 4, of the proposed framework are about 90.08% ± 5.31%, 0.22% ± 0.38%, 91.20% ± 3.77% and 92.62% ± 4.73%, respectively.


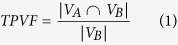



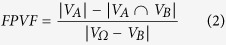



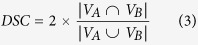



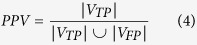


where 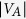, 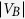 and 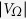 are the volume in the automatic segmentation result, ground truth and the total retina volume between the ILM and BM retinal layer, respectively. 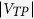 indicates the volume of the true positive segmented regions and 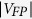 indicates the volume of the false positive segmented regions.

**Figure 4:**
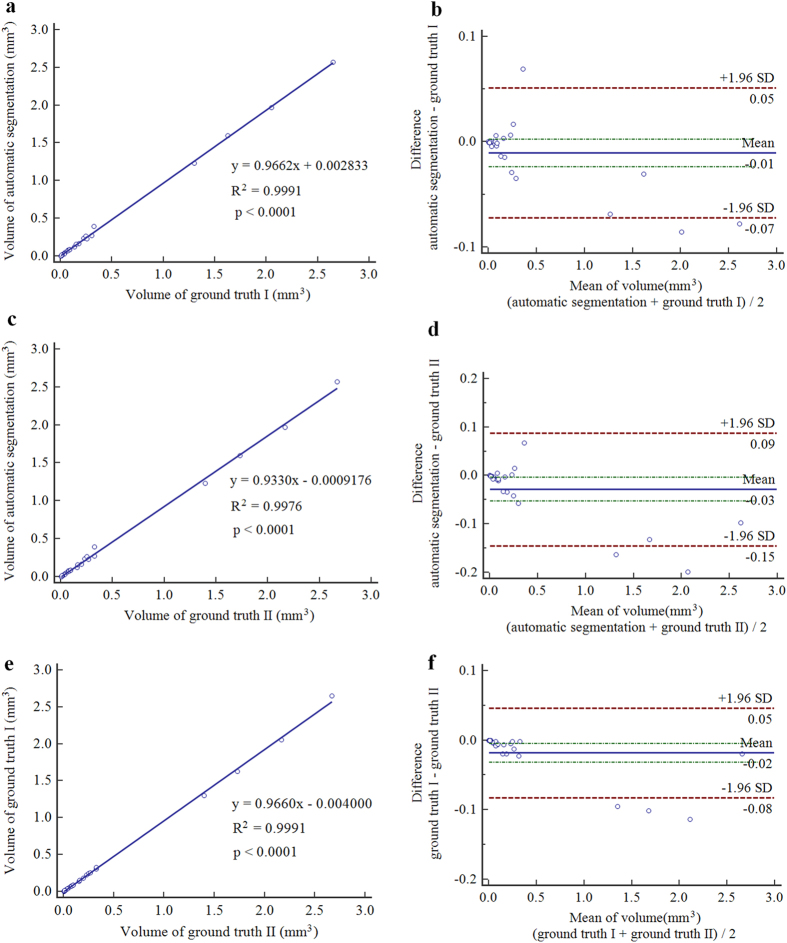
Correlation (**a**) and agreement (**b**) of the volume of retinal pigment epithelium detachment measured by automatic segmentation result and ground truth I. Correlation (**c**) and agreement (**d**) of the volume of retinal pigment epithelium detachment measured by automatic segmentation result and ground truth II. Correlation (**e**) and agreement (**f**) of the volume of retinal pigment epithelium detachment measured by ground truth I and II.

Comparing to the initial segmentation results, the final segmentation results show statically significantly higher FPVF, DSC and PPV (*p* < 0.05). Although the initial segmentation results get high TPVF, its segmentation results tend to have many false positive segmented regions. Therefore, both DSC and PPV are lower (see Table 1). Comparing to the segmentation results in ref. 15, the final segmentation results show slight higher TPVF (*p* = 0.326), and show statically significantly higher FPVF, DSC and PPV (*p* < 0.05). Figure 4 shows the linear regression analysis results and the Bland-Altman plots for the automatic segmentation results versus the ground truth I, the automatic segmentation results versus the ground truth II and the ground truth I versus the ground truth II, respectively. The figure demonstrates that: (1) A high correlation is obtained comparing the ground truth I to the ground truth II (R^2^ = 0.9991); (2) The automatic segmentation results have strong correlation with the ground truth I (R^2^ = 0.9991) and the ground truth II (R^2^ = 0.9976). Therefore, the automatic segmentation results from the proposed framework can replace the expert manual labeling; (3) The Bland-Altman figures reveal that the 95% limits of agreement are [−0.07, 0.05], [−0.15, 0.09] and [−0.08, 0.05] for the automatic segmentation results versus the ground truth I, the automatic segmentation results versus the ground truth II and the ground truth I versus the ground truth II, respectively.

## Discussion

In this paper, an automated framework is proposed for 3D serous PED segmentation in SD-OCT images, which effectively combines the multi-scale graph search, shape-constrained graph cut and mathematical morphology algorithm. The novelties of this work lie in: (1) an effective AdaBoost algorithm is applied to remove false positive segmented regions in the initial segmentation results; (2) the foreground and background seeds used in the shape-constrained graph cut method are obtained automatically, which makes the proposed framework automatic; (3) an adaptive structure elements based mathematical morphology method is applied to refine the automatic segmentation results. The proposed framework was tested on 25 SD-OCT data from 25 patients diagnosed with serous PED. In term of accuracy, the mean and standard deviation of TPVF, FPVF, DSC and PPV for the proposed framework are about 90.08% ± 5.31%, 0.22% ± 0.38%, 91.20% ± 3.77% and 92.62% ± 4.73%, respectively. Furthermore, the linear regression analysis shows a strong correlation comparing the automatic segmented PED to the ground truth I (R^2^ = 0.9991) and ground truth II (R^2^ = 0.9976), respectively.

One limitation of the proposed framework is that feature extraction is implemented in 2D. Because of the large variability in shape, size, position, and number of serous PED regions in different B-scans, extracting features is implemented B-scan by B-scan, which allows the abrupt change of serous PED regions properties.

The future work will be focused on three aspects to improve the framework. First, for large varied abnormities in retina, a more robust multi-scale graph search algorithm is needed to segment retinal layers, which allows the abrupt structure deformation in SD-OCT images. Second, the feature extraction can be extended to 3D to obtain more context information. Third, this work can be extended for more complicated pathological cases with RPE floor deformation or other co-existed fluid-filled abnormalities located above RPE floor, such as, choroidal neovascularization (CNV) segmentation.

In summary, an automated framework is proposed for 3D serous PED segmentation in SD-OCT images. As an efficient replacement of manual segmentation, the proposed framework can provide clinicians with accurate quantitative information, including shape, size and position of the PED regions, which can assist diagnosis and treatment.

## Methods

### Method overview

**Figure 5:**
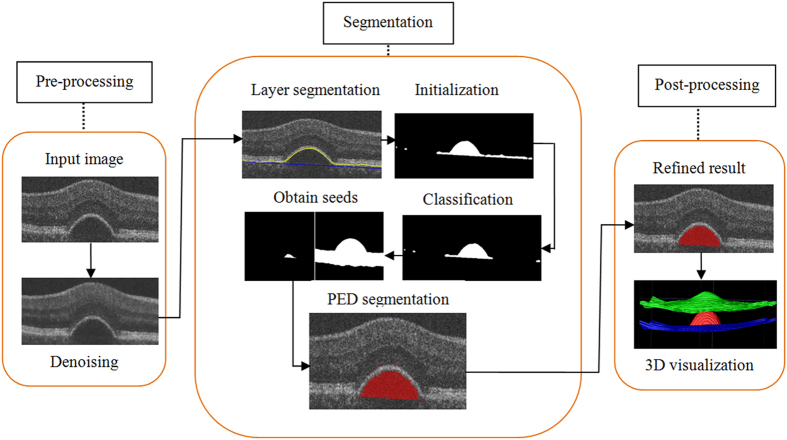
The flowchart of the proposed framework.

The proposed framework includes three major parts: pre-processing, segmentation and post-processing (see the flowchart in Fig. 5). For pre-processing, an improved curvature anisotropic diffusion filter is applied to remove speckle noise. For segmentation, first, the multi-scale graph search method is applied to segment abnormal retinal layers associated with RPE floor deformation. During this process, BM is estimated with convex hull algorithm; second, 62 features are extracted for classification and then an AdaBoost classifier is applied to remove false positive initial segmented regions; third, the serous PED regions are segmented by using the shape-constrained graph cut method, in which foreground and background seeds are obtained automatically. For post-processing, an adaptive structure elements based mathematical morphology method is applied to refine the automatic segmentation results.

### Denoising

**Figure 6:**
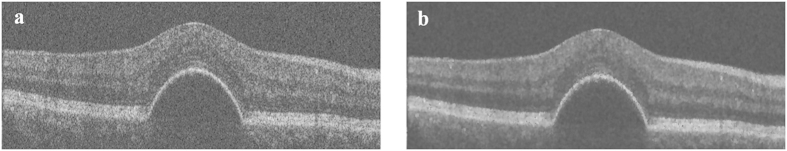
A cross sectional image before (**a**) and after (**b**) denoising.

Speckle noise and artifacts are the dominant factors for image quality degrading in SD-OCT scans. For poor quality retinal images, the efficiency and accuracy of the segmentation algorithm can decrease greatly. The curvature anisotropic diffusion filter can remove speckle noise and artifacts effectively and preserve the image boundary well at the same time. Comparing to the typical curvature anisotropic diffusion filtering method, a modified curvature diffusion equation (MCDE) is defined as follows^26^,^27^.


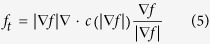


where 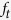 is the output image, 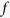 is the input image, 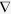 is the gradient operation, and 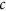 is the conductance function. The conductance represents the gradient magnitude of each pixel in the image, and it is used to reduce the diffusion strength of edge pixels. The denoised result is shown in Fig. 6 (b). Comparing to the original image in Fig. 6 (a), the speckle noise is suppressed effectively while the edges are preserved well, which helps abnormal retinal layers segmentation.

### Multi-scale graph search

The 3D graph search algorithm proposed by Li *et al.* for optimal surface segmentation^28^, has been used for normal retinal layers segmentation. However, for abnormal retina with PED, the serious deformation of RPE floor and blurred boundary of retinal layers can cause great difficulties for retinal layers segmentation^15^. In order to solve this problem, in this paper, a multi-scale graph search algorithm is applied for serous PED retinal layers segmentation. The 3D SD-OCT image is down-sampled by a factor of 2 twice in the vertical direction to form three scales: the lowest resolution, the medium resolution and the highest resolution. Then, a single surface graph search method is applied in three scales from low resolution to high resolution scale. The surface location in the lower resolution can serve as a position constraint for optimal surface location in the higher resolution scale. The multi-scale method greatly reduces the searching space of the optimization step and therefore enhances the segmentation efficiency. Three retinal surfaces are segmented by using single surface graph search method, namely, the inner limited membrane (ILM), roof of photoreceptor ellipsoid zone and RPE floor. The ILM and roof of photoreceptor ellipsoid zone have the dark-to-bright transition, while RPE floor has the bright-to-dark transition. First, with the most prominent edge in the SD-OCT image, the ILM is segmented, because its characteristic is not severely affected by the presence of serous PED. Second, the roof of photoreceptor ellipsoid zone and RPE floor are segmented respectively based on the position constraints provided by previous segmented retinal surfaces. Both roof of photoreceptor ellipsoid zone and RPE floor tend to have abrupt shape change with arch-like shape for serous PED. Therefore, a larger smoothness constraint should be set to capture those abrupt shape changes, which is different from normal retinal surfaces segmentation^15^,^16^.

**Figure 7:**
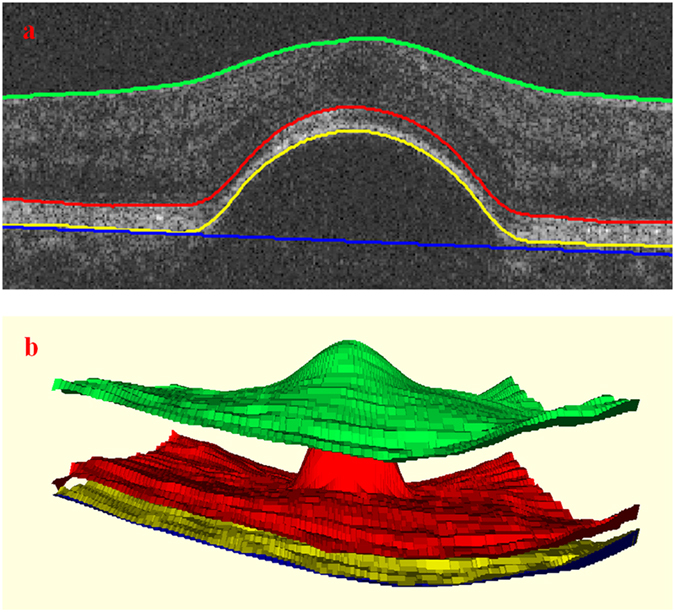
The result of retinal layers segmentation. (**a**) The 2D visualization on a B-scan. The green line, red line, yellow line and blue line represent the internal limiting membrane, roof of photoreceptor ellipsoid zone, RPE floor and Bruch’s membrane, respectively; (**b**) The 3D visualization of four segmented retinal surfaces.

**Figure 8:**
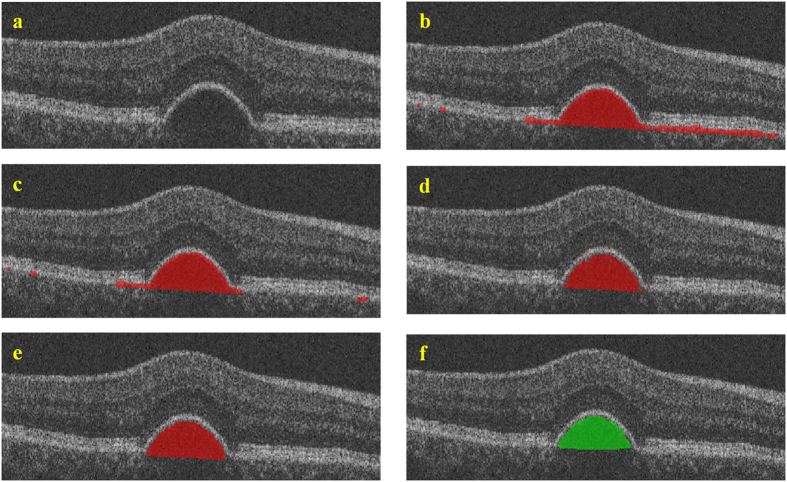
The experimental results at different phrases. (**a**) is one example of the original images, (**b**) is one example of the initial segmentation results, (**c**) is one example of the classification results, (**d**) is one example of the segmentation results obtained by using the shape-constrained graph cut method, (**e**) is one example of the morphological operation results, and (**f**) is one example of the ground truths.

Finally, BM under RPE floor, which is not distinct in SD-OCT images, is estimated from the RPE floor by finding the lower boundary of its convex hull. For a finite point set P = {p_1_, p_2_, …, p_k_, …, p_n_}, the convex hull of P is a minimum convex polygon that wraps P. Figure 7 shows the result of retinal layers segmentation with four retinal surfaces and its 3D visualization. After getting the four retinal surfaces, the region between the RPE floor and BM is defined as the initial segmentation result for serous PED. Figure 8 (b) shows one example of the initial segmentation results by overlaying the segmented result on the original image, in which the red regions are the segmented serous PED regions. Some initial segmentation results may not be accurate due to the possible error in RPE floor segmentation and BM estimation resulting from the blurred retinal layers boundary in the original images. Some false positive segmented regions can be removed by latter classification method.

### Feature extraction

Robust features are critical for successfully segmenting abnormal structures in medical images^29^,^30^. In this paper, because of the noise, artifact, blurred boundary and serious deformation of abnormal retinal structures, extracting useful features is a difficult task. Furthermore, variability in shape, size, position, number and texture properties of serous PED further makes the search for robust features more complicated. The shape, intensity and texture features are extracted for serous PED in SD-OCT images. The features are extracted based on the initial segmentation results for each segmented region. Feature 1 to 17 are the area, perimeter, major axis length, minor axis length, major axis length/minor axis length, perimeter/area, eccentricity, orientation, Euler number, the number of bright pixels in the segmented region, the diameter of a circle with the same area as the segmented region, solidity, extent, convex hull area, max intensity, min intensity and mean intensity. Feature 18 to 21 are distance from the centroid to retinal surface ILM, roof of photoreceptor ellipsoid zone, RPE floor and BM. Feature 22 to 28 are the coordinates and intensity of the centroid, and the coordinates of the min rectangle wrapping the segmented region. Feature 29 to 52 are the coordinates and intensity of the extremum points in eight different directions. Feature 53 to 62 are regional mean of two eigenvalues of the Hessian matrix at scale 1, 3, 6, 9 and 14. In this paper, feature extraction is implemented on 2D slices automatically, allowing the variability in shape, size, position and number of serous PED regions in different B-scans.

### Classification

In this paper, only the serous PED region and background will be distinguished. Therefore it is a two-class classification problem.

An AdaBoost classifier is applied to distinguish serous PED object from background. All training samples will be set with an original weight respectively at the beginning. The original weight will be updated with the principle that the weight of wrong classified sample increases while the weight of correct classified sample decreases for the next training^31^. Finally, a high-performance strong classifier will be trained by combining the weighted value of a set of weak classifiers. The AdaBoost classification algorithm is described as Pseudo-code 1.

Figure 8 (c) shows one example of the classification results. Some false positive segmented regions are removed by the trained AdaBoost classifier comparing to the initial segmentation results in Fig. 8 (b). After removing the false positive segmented regions, the refined initial segmentation results will be used to obtain foreground and background seeds automatically, which help to segment serous PED by using the shape-constrained graph cut algorithm.

Pseudo-code 1. AdaBoost classification algorithm:

Input: The initial segmentation results and the extracted features.

Begin

1. Assign an original weight for each training sample;
2. Train a weak classifier and compute the error rate and weighted value of the weak classifier;
3. Update the weight of training sample with the principle that the weight of wrong classified sample increases and the weight of correct classified sample decreases;
4. Repeat 2) to get N weak classifiers;
5. Combine the weighted value of N weak classifiers to constitute a strong classifier.

End

Output: The refined initial segmentation results.

### Shape-constrained graph cut

The graph cut method has been used widely for the region object segmentation^32^,^33^,^34^,^35^,^36^, which is formulated as an energy minimization problem by using min-cut/max-flow algorithm^37^,^38^,^39^. For the graph cut method, an important step is to obtain foreground and background seeds. Traditionally, the seeds are labeled manually. In this paper, the seeds are obtained automatically by the mathematical morphology operations based on the refined initial segmentation results. The erosion operation is applied to obtain foreground seeds, and the dilation operation is applied to obtain background seeds. More details about the mathematical morphology method will be introduced in section *Mathematical morphology*. In this paper, the cost function for the shape- constrained graph cut has three parts, including the region term, the boundary term and the shape term, defined as follows:


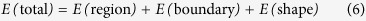


where 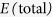 is the total cost, 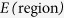 is the cost associated with the voxel intensity value, 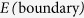 is the cost associated with the intensity gradient, and 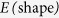 is the cost associated with the shape of serous PED. Comparing to the typical graph cut cost function, the shape term is added in this paper.


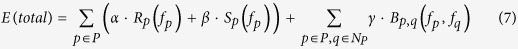



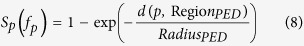


where 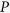 and 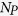 are the set of pixels, 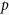 and 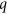 are pixel in 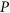 and 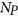 respectively, and 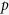 is the neighborhood of 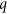. *f*_*p*_ and *f*_*q*_ are label assigned to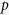 and 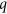 respectively. *R*_*p*_(*f*_*p*_), the region term, is a cost based on the voxel intensity value. *B*_*q*,*p*_(*f*_*p*_, *f*_*q*_), the boundary term, is the cost based on the gradient of the image intensity. *S*_*p*_(*f*_*p*_), the shape term, is the cost based on the shape of serous PED. 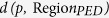 is a distance from pixel 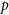
to the PED region. The linear time method in ref. 40 is applied to compute 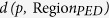, and defining that if 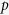 belongs to 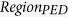, then 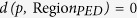. 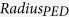 is the radius of a circle that just encloses 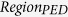. The 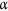, 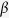 and 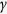 are three weights for *R*_*p*_(*f*_*p*_), *S*_*p*_(*f*_*p*_) and *B*_*q*,*p*_(*f*_*p*_, *f*_*q*_) respectively, satisfying 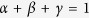. Only 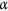 and 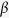 are estimated using the gradient descent method in ref. 41 and set 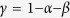. In this paper, the shape-constrained graph cut method is implemented after non-linear brightness curve transform based on the retinal layers segmentation results in SD-OCT images^42^. Figure 8 (d) shows one example of the segmented serous PED regions obtained by the shape-constrained graph cut method and the red regions are the segmented regions.

### Mathematical morphology

The mathematical morphology is applied for obtaining foreground seeds, background seeds and refining the automatic segmentation results in this paper. The critical step of mathematical morphology is to choose appropriate structure element (SE), which decides the performance of morphological operations. The shape and size are two important properties of SE. The shape of SE can be defined as rectangle, disk, diamond or ball etc, and its size also has multiple choices. Single SE is not enough for abnormal retinal images processing with serous PEDs, which have various shapes, sizes and positions. In this paper, a multiple SE morphology method is applied to refine the serous PED automatic segmentation result, which allows the variability in shape, size and position of serous PED. The basic principle of multiple SE is to construct different SE with different size while preserving the same shape based on the segmented PED regions. The shape and size of SE are designed based on the extracted shape features, such as, the major axis length, minor axis length and area etc. In this paper, the shape of SE is defined as ball and its radius is defined as follows:


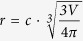


where 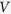 is the volume of the segmented PED region, 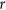 is the radius of SE, and *c* is a coefficient. By adaptively choosing the radius of SE, the erosion operation is applied to obtain foreground seeds (*c* = 0.143), and the dilation operation is applied to obtain background seeds (*c* = 1.143) based on the refined initial segmentation results. The obtained foreground and background seeds are used in the shape-constrained graph cut method.

Because the abnormal retinal images tend to have blurred boundary and serious deformation in roof of photoreceptor ellipsoid zone and RPE floor, the automatic segmentation results may have some spurs, grooves or isolated points. In this paper, the reconstruction operation and closing operation are implemented to refine the automatic segmentation results obtained from the shape-constrained graph cut method^43^. The SE is also set as ball with adaptive radiuses as in Equation (9) for reconstruction operation (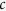 = 0.200) and closing operation (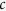 = 0.700). Figure 8 (e) shows one example of the morphology operation results. Comparing to Fig. 8 (d), some spurs and isolated points are removed, and some grooves are filled up.

### Evaluation of segmentation performance

In order to evaluate the segmentation performance, the automatic segmentation result is compared to ground truth, the initial segmentation result and the segmentation result in ref. 15. Paired t-test is applied to compute the *p* value and a *p* value less than 0.05 is considered statistically significant. Four indices are chosen to assess the automatic segmentation performance, including TPVF, FPVF, DSC and PPV.

## Additional Information

**How to cite this article**: Sun, Z. *et al.* An automated framework for 3D serous pigment epithelium detachment segmentation in SD-OCT images. *Sci. Rep.* **6**, 21739; doi: 10.1038/srep21739 (2016).

## Figures and Tables

**Figure 1:**
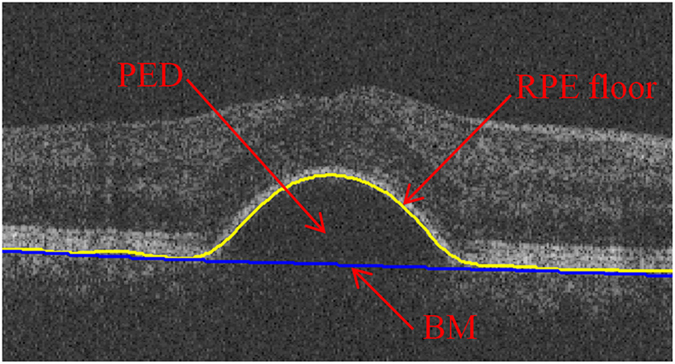
A cross-sectional (B-scan) image of spectral domain optical coherence tomography with serous pigment epithelium detachment.

**Table 1:** Mean ± standard deviation of the serous pigment epithelium detachment segmentation result using the ground truth I as the reference.

	True positive volume fraction (%)	False positive volume fraction (%)	Dice similarity coefficient (%)	Positive predictive value (%)
Initial result	96.00 ± 5.06	1.95 ± 2.30	59.00 ± 28.05	48.94 ± 29.05
Result in ref. 15	87.10 ± 21.70	0.37 ± 0.54	83.75 ± 20.22	81.20 ± 20.20
Ground truth II	97.46 ± 2.61	0.21 ± 0.35	94.73 ± 2.12	92.20 ± 2.76
Proposed method	90.08 ± 5.31	0.22 ± 0.38	91.20 ± 3.77	92.62 ± 4.73

## References

[b1] Huang D. *et al.* Optical coherence tomography. Science 254, 1178–1181 (1991). 1957169 10.1126/science.1957169 PMC4638169

[b2] Cukras C. *et al.* Optical coherence tomography-based decision making in exudative age-related macular degeneration: comparison of time vs spectral- domain devices. Eye 24, 775–783 (2010). 19696804 10.1038/eye.2009.211 PMC3016921

[b3] Zayit-Soudry S., Moroz I. & Loewenstein A. Retinal pigment epithelial detachment. Surv Ophthalmol 52, 227–243 (2007). 17472800 10.1016/j.survophthal.2007.02.008

[b4] Lommatzsch A., Helmes B., Gutfleisch M. & Spital G. Serous pigment epithelial detachment in age-related macular degeneration: comparison of different treatments. Eye 23, 2163–2168 (2009). 19197318 10.1038/eye.2008.425

[b5] Mrejen S., Sarraf D., Mukkamala S. K. & Freund K. B. Multimodal imaging of pigment epithelial detachment: a guide to evaluation. Retina 33, 1735–1762 (2013). 23873168 10.1097/IAE.0b013e3182993f66

[b6] Keane P. A. *et al.* Evaluation of age-related macular degeneration with optical coherence tomography. Surv Ophthalmol 57, 389–414 (2012). 22898648 10.1016/j.survophthal.2012.01.006

[b7] Garvin M. K. *et al.* Intraretinal layer segmentation of macular optical coherence tomography images using optimal 3-D graph search. IEEE Trans Med Imaging 27, 1495–1505 (2008). 18815101 10.1109/TMI.2008.923966 PMC2614384

[b8] Garvin M. K. *et al.* Automated 3-D intraretinal layer segmentation of macular spectral-domain optical coherence tomography images. IEEE Trans Med Imaging 28, 1436–1447 (2009). 19278927 10.1109/TMI.2009.2016958 PMC2911837

[b9] Song Q. *et al.* Optimal multiple surface segmentation with shape and context priors. IEEE Trans Med Imaging 32, 376–386 (2013). 23193309 10.1109/TMI.2012.2227120 PMC4076846

[b10] Kafieh R., Rabbani H., Abràmoff M. D. & Sonka M. Intra-retinal layer segmentation of 3D optical coherence tomography using coarse grained diffusion map. Med Image Anal 17, 907–928 (2013). 23837966 10.1016/j.media.2013.05.006 PMC3856938

[b11] Lang A. *et al.* Retinal layer segmentation of macular OCT images using boundary classification. Biomed Opt Express 4, 1133–1152 (2013). 23847738 10.1364/BOE.4.001133 PMC3704094

[b12] Lee K. M. *et al.* Segmentation of the optic disc in 3-D OCT scans of the optic nerve head. IEEE Trans Med Imaging 29, 159–168 (2010). 19758857 10.1109/TMI.2009.2031324 PMC2911797

[b13] Chen X. *et al.* 3D segmentation of fluid-associated abnormalities in retinal OCT: probability constrained graph-search-graph-cut. IEEE Trans Med Imaging 31, 1521–1531 (2012). 22453610 10.1109/TMI.2012.2191302 PMC3659794

[b14] Chen H. *et al.* Quantitative analysis of retinal layers’ optical intensities on 3D optical coherence tomography for central retinal artery occlusion. Sci Rep 5, 9269 (2015). 25784298 10.1038/srep09269 PMC4363859

[b15] Shi F. *et al.* Automated 3-D retinal layer segmentation of macular optical coherence tomography images with serous pigment epithelial detachments. IEEE Trans Med Imaging 34, 441–452 (2015). 25265605 10.1109/TMI.2014.2359980 PMC13698588

[b16] Sun Z., Shi F., Xiang D., Chen H. & Chen X. Automated segmentation of serous pigment epithelium detachment in SD-OCT images. SPIE Medical Image 2015 (2015). 10.1038/srep21739 PMC4761989 26899236

[b17] Fernández D. C. Delineating fluid-filled region boundaries in optical coherence tomography images of the retina. IEEE Trans Med Imaging 24, 929–945 (2005). 16092326 10.1109/TMI.2005.848655

[b18] Ahlers C. *et al.* Automatic segmentation in three-dimensional analysis of fibrovascular pigment epithelial detachment using high-definition optical coherence tomography. Brit J Ophthalmol 92, 197–203 (2007). 17965102 10.1136/bjo.2007.120956

[b19] Quellec G. *et al.* Three-dimensional analysis of retinal layer texture: identification of fluid-filled regions in SD-OCT of the macula. IEEE Trans Med Imaging 29, 1321–1330 (2010). 20363675 10.1109/TMI.2010.2047023 PMC2911793

[b20] Dolejši M., Abràmoff M. D., Sonka M. & Kybic J. Semi-automated segmentation of symptomatic exudate-associated derangements (SEADs) in 3D OCT using layer segmentation. Biosignal (2010).

[b21] Gregori G., Wang F. & Rosenfeld P. J. Spectral domain optical coherence tomography imaging of drusen in nonexudative age-related macular degeneration. Ophthalmology 118, 1373–1379 (2011). 21388687 10.1016/j.ophtha.2010.11.013 PMC3129493

[b22] Penha F. M. *et al.* Quantitative imaging of retinal pigment epithelial detachments using spectral-domain optical coherence tomography. Am J Ophthalmol 153, 515–523 (2012). 22030354 10.1016/j.ajo.2011.08.031

[b23] Wilkins G. R., Houghton O. M. & Oldenburg A. L. Automated segmentation of intraretinal cystoid fluid in optical coherence tomography. IEEE T Biomed Eng 59, 1109–1114 (2012). 10.1109/TBME.2012.2184759 PMC3725742 22271827

[b24] Zheng Y. *et al.* Computerized assessment of intraretinal and subretinal fluid regions in spectral-domain optical coherence tomography images of the retina. Am J Ophthalmol 155, 277–286 (2013). 23111180 10.1016/j.ajo.2012.07.030

[b25] Ding W., Young M. & Bourgault S. Automatic detection of subretinal fluid and sub-retinal pigment epithelium fluid in optical coherence tomography images. *35th Annual International Conference of the IEEE EMBS* (2013). 10.1109/EMBC.2013.6611265 24111452

[b26] Whitaker R. T. & Xue X. Variable-conductance, level-set curvature for image denoising. Proceeding of International Conference on Image Processing 3, 142–145 (2001).

[b27] Yang Y., Lin P. & Zheng C. An efficient statistical method for segmentation of single-channel brain MRI. *Proceedings of the Fourth International Conference on Computer and Information Technology* (2004).

[b28] Li K., Wu X., Chen D. Z. & Sonka M. Optimal surface segmentation in volumetric images—a graph-theoretic approach. IEEE T Pattern Anal 28, 119–134 (2006). 10.1109/TPAMI.2006.19 PMC2646122 16402624

[b29] Xu Y., Sonka M., McLennan G., Guo J. & Hoffman E. A. MDCT-based 3-D textural classification of emphyema and early smoing related lung pathologies. IEEE Trans Med Imaging 25, 464–475 (2006). 16608061 10.1109/TMI.2006.870889

[b30] Ahmed S. & Iftekharuddin K. M. Efficacy of texture, shape, and intensity feature fusion for posterior-fossa tumor segmentation in MRI. IEEE T Inf Technol B 15, 206–213 (2011). 10.1109/TITB.2011.2104376 21216716

[b31] Gao L., Kou P., Gao F. & Guan X. AdaBoost regression algorithm based on classification-type loss. Proceedings of the 8th World Congress on Intelligent Control and Automation, 682–687 (2010).

[b32] Chen X. & Bagci U. 3D automatic anatomy segmentation based on iterative graph-cut-ASM. Med Phys 38, 4610–4622 (2011). 21928634 10.1118/1.3602070 PMC3298556

[b33] Chen X., Udupa J. K., Bağcı U., Zhuge Y. & Yao J. Medical image segmentation by combining graph cut and oriented active appearance models. IEEE T Image Process 21, 2035–2046 (2012). 10.1109/TIP.2012.2186306 PMC5548181 22311862

[b34] Chen X. *et al.* A framework of whole heart extracellular volume fraction estimation for low dose cardiac CT images, IEEE T Inf Technol B 16, 842–851 (2012). 10.1109/TITB.2012.2204405 PMC3491075 22711778

[b35] Chen X., Udupa J. K., Alavi A. & Torigian D. A. GC-ASM: synergistic integration of graph-cut and active shape model strategies for medical image segmentation. Comput Vis Image Und 117, 513–524 (2013). 10.1016/j.cviu.2012.12.001 PMC3622953 23585712

[b36] Ju W., Xiang D., Zhang B., Kopriva I. & Chen X. Random walk and graph cut for co-segmentation of lung tumor on PET-CT images, IEEE T Image Process 24, 5854–5867 (2015). 10.1109/TIP.2015.2488902 26462198

[b37] Kolmogorov V. & Zabih R. What energy function can be minimized via graph cuts? IEEE T Pattern Anal 26, 147–159 (2004). 10.1109/TPAMI.2004.1262177 15376891

[b38] Boykov Y. & Kolmogorov V. An experimental comparison of min-cut/max-flow algorithms for energy minimization in vision. IEEE T Pattern Anal 26, 1124–1137 (2004). 10.1109/TPAMI.2004.60 15742889

[b39] Boykov Y. & Funka-Lea G. Graph cuts and efficient N-D image segmentation. Int J Comput Vision 70, 109–131 (2006).

[b40] Ciesielski K. C., Chen X., Udupa J. K. & Grevera G. J. Linear time algorithms for exact distance transform. J Math Imaging and Vis 39, 193–209 (2010).

[b41] Snyman J. A. Practical mathematical optimization: an introduction to basic optimization theory and classical and new gradient-based algorithms. (Springer, 2005).

[b42] Xu X., Lee K., Zhang L., Sonka M. & Abràmoff M. D. Stratified sampling voxel classification for segmentation of intraretinal and subretinal fluid in longitudinal clinical OCT data. IEEE Trans Med Imaging 34, 1616–1623 (2015). 10.1109/TMI.2015.2408632 PMC5750134 25769146

[b43] Miri M. S. & Mahloojifar A. Retinal image analysis using curvelet transform and multistructure elements morphology by reconstruction. IEEE T Biomed Eng 58, 1183–1192 (2011). 10.1109/TBME.2010.2097599 21147592

